# Polymer Optical Fiber Sensors in Healthcare Applications: A Comprehensive Review

**DOI:** 10.3390/s19143156

**Published:** 2019-07-18

**Authors:** Arnaldo G. Leal-Junior, Camilo A.R. Diaz, Letícia M. Avellar, Maria José Pontes, Carlos Marques, Anselmo Frizera

**Affiliations:** 1 Mechanical Engineering Department, Federal University of Espírito Santo, Espírito Santo 29075-910, Brazil; 2 Graduate Program in Electrical Engineering, Federal University of Espírito Santo, Espírito Santo 29075-910, Brazil; 3 Department of Physics and I3N, University of Aveiro, Campus Universitário de Santiago, 3810-193 Aveiro, Portugal

**Keywords:** Polymer optical fiber, fiber bragg gratings, optical fiber sensors, wearable robots, wearable sensors, healthcare

## Abstract

Advances in medicine and improvements in life quality has led to an increase in the life expectancy of the general population. An ageing world population have placed demands on the use of assistive technology and, in particular, towards novel healthcare devices and sensors. Besides the electromagnetic field immunity, polymer optical fiber (POF) sensors have additional advantages due to their material features such as high flexibility, lower Young’s modulus (enabling high sensitivity for mechanical parameters), higher elastic limits, and impact resistance. Such advantages are well-aligned with the instrumentation requirements of many healthcare devices and in movement analysis. Aiming at these advantages, this review paper presents the state-of-the-art developments of POF sensors for healthcare applications. A plethora of healthcare applications are discussed, which include movement analysis, physiological parameters monitoring, instrumented insoles, as well as instrumentation of healthcare robotic devices such as exoskeletons, smart walkers, actuators, prostheses, and orthosis. This review paper shows the feasibility of using POF sensors in healthcare applications and, due to the aforementioned advantages, it is possible to envisage a further widespread use of such sensors in this research field in the next few years.

## 1. Introduction

As an emerging sensor technology, optical fiber sensors present the intrinsic advantages of lightweight, compactness, chemical stability, immunity to electromagnetic field, and multiplexing capabilities [1]. These advantages make optical fiber sensors an intrinsically safe technology for industrial [2], medical [3], and structural health monitoring [4] applications. Additionally, optical fiber sensors are employed on the measurement of several parameters like angle [5], refractive index [6], temperature [7], humidity [8], acceleration [9], pressure [10], breathing rate [11], and oxygen saturation [12]. In addition, the features of optical fibers enable their embedment in textiles for sensing applications [11,13] and also the creation of optical fiber-based textiles, the so-called photonics textiles [12], as well as the integration on composite laminates [14], metals by welding process [15], concrete [16], and even in three dimensional (3D)-printed structures [17].

Concurrently with the optical fiber sensors advances, the demands for healthcare devices are continuously increasing, especially due to the population ageing [18]. In addition, advances in electronics, materials processing, and data transmission lead to a new generation of robotic devices [19], wearable sensors [20], and cloud services for healthcare [21]. As novel healthcare devices and applications emerge, more demands are placed on the sensors’ performance, since robust control strategies for wearable robots need a reliable sensor system [22]. In addition, with the components miniaturization, the sensor system must be as flexible and compact as possible [23]. For intrusive sensor systems, the demands are even higher since the sensor system needs all the aforementioned requirements and the biocompatibility [24]. Thus, the sensor systems are in constant development in order to cope with required performance of novel wearable systems and devices.

Conventionally, the sensor systems use electronic or electromechanical sensors, which can present some drawbacks such as sensitivity to misalignments, electromagnetic fields sensitivity (undesired when electric actuators are activated in wearable robots), lower compactness, necessity of frequent calibration and, in some cases, hysteresis and drift on the sensor response [25]. The disadvantages of electronic sensors are especially undesirable in soft wearable robotics. In this case, the robotic structure and actuators have flexible materials in their composition in order to achieve lower weight and higher compliance with the user [26]. Thus, novel customized actuators [27] and robots are proposed [28], where there is also the possibility to design a robot with geometries and actuators especially designed for a specific user in the so-called human-in-the-loop design [26]. Considering the soft robot structures, the sensors are constantly submitted to large strains or deflections of the structure, which can inhibit the application of most conventional sensors [23] since only flexible electronic sensors would be able to operate in such conditions [23]. However, such sensors generally need complex manufacturing techniques.

Considering the increasing demands on sensor systems in conjunction with the fast development of optical fiber sensors, many sensors systems for healthcare and medical devices using the optical fiber technology have been proposed, which are summarized in some review papers [3,29,30] and book chapters [31]. Some applications of the optical fiber sensors include study of bones decalcification and strain distribution [32], evaluation of intervertebral disks [33], dental splints [34], cardiac monitoring [35], and biosensors for pathologies detection [3]. Furthermore, many pressure sensors were proposed for intramuscular, intracranial, and intra-articular pressure assessment, as summarized in reference [36]. Applications of optical fiber sensors also include pressure assessment in prosthesis sockets [37] and strain distribution on the prosthesis [38] as well as on the investigation of actuator dynamics in orthosis [39]. For movement analysis, optical fiber sensors have been applied in joint angle [40] and plantar pressure monitoring [41].

The biomedical and healthcare applications (all applications aforementioned) were initially proposed with silica optical fibers due to their higher use leading to a higher maturity than with other types of optical fibers. There are two major types of optical fibers, silica and polymer optical fibers (POFs). Besides their higher optical power attenuation when compared with silica fibers, POFs present advantages over silica fibers related to their material features. Such advantages include higher flexibility, lower Young’s modulus, higher strain limits, fracture toughness, and impact resistance [1]. Furthermore, POFs enable a safer operation in smart textiles and intrusive applications, since silica fibers can break more easily than POFs and present a risk for the users when there is a breakage due to the glass punctures that might cause injuries [11].

The POF’s high flexibility enables its embedment in soft structures, which makes it a suitable option for the instrumentation of such novel wearable robots. It is worth noting that 3D printing is one of the technologies that have enabled the development of such novel wearable flexible systems with lower relative, which is an additive layer manufacturing process, where a hot or melted polymer is injected layer-by-layer [42]. The materials generally used in 3D printing are polylactic acid (PLA) or acrylonitrile butadiene styrene (ABS) with the possibility of using flexible materials such as thermoplastic polyurethane (TPU). In this case, optical fiber sensors are embedded in 3D-printed structures for structural health monitoring (SHM) [43] and plantar pressure sensing platforms [44].

These advantages of POFs in conjunction with developments on POFs materials processing, fiber connectorization and sensors production have pushed the POF sensor technology towards many commercial applications in different fields, as summarized in reference [45], where the developments related to FBGs in POFs and their many applications were summarized and discussed in detail. This new scenario in POF sensors has enabled the development of many healthcare devices using POFs as the sensing element with the potential for commercialization and further widespread use. In order to address these new POF sensor systems for healthcare, this paper presents a review of POF sensors in healthcare devices, where such the applications include movement analysis, wearable robot’s instrumentation, novel assistive devices, and assessment of physiological parameters.

The remainder of this paper is divided as follows. In Section 2, an overview of the healthcare scenario as well as the conventional sensors used in healthcare devices are discussed. Moreover, the trends on healthcare applications are also discussed and placed in context with the development of POF sensors. Section 3 presents a discussion of POF sensor technologies, which covers the development of novel materials for POFs in applications with different requirements e.g., high strain and temperature limits, flexibility, fracture toughness, and biocompatibility. After discussing the current scenario and trends in healthcare as well as the developments on POF sensors, Section 4 discusses the applications of POF sensors in healthcare, where such applications cover many aspects of healthcare such as wearable robots, assistive devices, wearable sensors, and physiological parameters monitoring. Finally, conclusions and final remarks are depicted in Section 5.

## 2. Healthcare: Current Scenario and Future Trends

**Figure 1:**
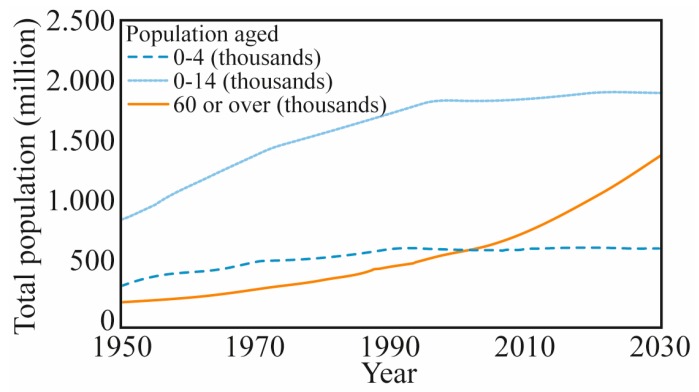
World population ageing throughout the years and predictions for the next 10 years, adapted from [18].

The continuous increase of the life expectancy due to improvements in the quality of life and advances in medicine as well as the increasing interest in family planning and birth control policies in developed countries have led to a rapid and progressive increase on ageing of the world’s population [18]. According to the World Health Organization, by 2020 the number of elderly people (over 60 years) will be higher than the children below 5 years [46] (Figure 1). The population ageing also results in an increase of clinical conditions that affect the human locomotion such as stroke, spinal cord injury, Parkinson’s disease, and weakness of the skeletal muscles [19]. This scenario has pushed the boundaries for novel therapeutic methods and assistance devices for patients with locomotor impairment, which also result in the development of novel devices with the aim of monitoring parameters for human health assessment [21].

In order to offer independence and attenuate the effects of the human gait disorders, different gait assistance devices have been proposed throughout the years, e.g., prostheses [47], exoskeletons [48], orthosis [49], and smart walkers (SWs) [50], with the expectation of an even higher increase in their use. The SW is generally used as a supporting device in the patients bipedestation, which aids in their balance and thus, improves mobility [50]. SWs present actuators and electronic components aiming to provide better assistance to the users, where the functionalities of such devices include autonomous control, with the possibility of shared or manual control as well, sensorial feedback, higher safety, and the possibility of monitoring the user’s state [51]. Among the wearable robotic devices for gait rehabilitation, lower limb exoskeletons show advantages over conventional rehabilitation therapies related to their higher repeatability in rehabilitation exercises, possibility of treatment customization, and quantitative feedback of the patient’s recovery [52]. In addition, wearable robots control strategies for human–robot cognitive interaction enable the use of exoskeletons as assistance devices for daily activities, which include the gait assistance [53].

In order to achieve their functionalities, exoskeletons highly rely on sensor systems, which are generally potentiometers and encoders for the joint angles assessment that have to be carefully attached to the device’s joints due to their sensitivity to misalignments [25]. For this reason, these sensors need mechanical supports precisely attached to the robot, which result in a less compact system [54]. Another commonly employed sensor technology is based on inertial measurement units (IMUs), but they need regular calibration [55]. In addition, the high sensitivity to electromagnetic fields can be regarded as an important disadvantage in wearable robots applications in which electric actuators are constantly activated [54]. Similarly, the electronic strain gauges employed in the assessment of human–robot force/torque interaction and actuator dynamics also have sensitivity to electromagnetic fields, need frequency calibration, and require a careful attachment to the structure, where the strain/force will be measured [56].

**Figure 2:**
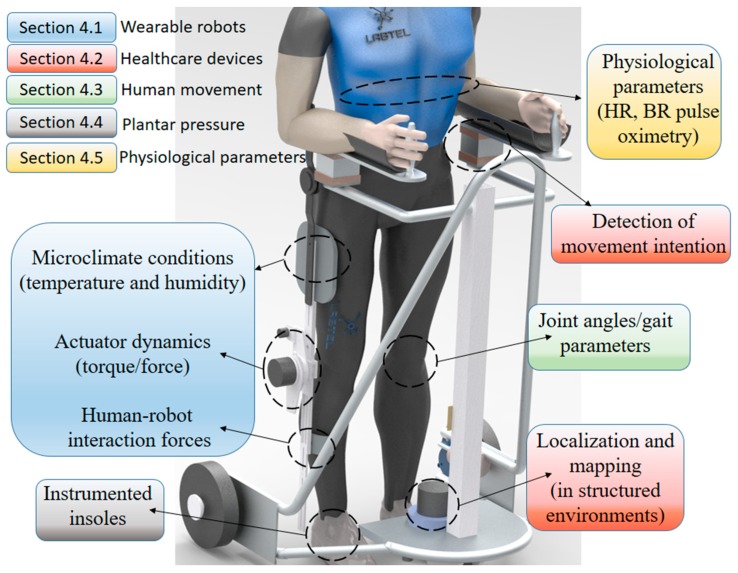
Wearable healthcare devices and parameters for POF sensor applications. The figure also indicates which section each application is in.

Figure 2 shows a schematic representation of the robotic devices (lower limb exoskeleton and SW) and monitored parameters, which not only outline the aforementioned parameters and devices, but also show the sensors discussed throughout this paper with different POF sensing approaches.

The microclimate conditions of wearable devices are the temperature and humidity measured on the interface between the device and its user. The assessment of these parameters is important, since it is related to pressure ulcers and the patient’s comfort while using the device [25]. However, the electronic sensors used for this purpose can present low linearity or dynamic range [25]. In some cases, they have low accuracy and stability as well [25].

Different control strategies for human–robot interaction proposed for SW are summarized in reference [51] and require different types of sensors. The sensors for the SW instrumentation can be divided into four main groups: force sensors for upper limbs strategies, sensors for kinematic assessment of the gait, odometry sensors, and sensors for environment interaction of the SW, where the device tracks the human position to minimize the differences in their orientations and distances [57]. Considering the system of Figure 2, the orientation is obtained through IMUs positioned in the user’s hip and the cadence is obtained with a laser range finder (LRF) that detects the legs of the user, whereas two 3D force sensors are applied on the detection of the user’s movement intention [58]. Moreover, a light detection and ranging (LIDAR) can be used on the environment localization and mapping.

The monitored parameters for human health assessment include foot plantar pressure, which provides important data regarding the human health condition [59]. With the plantar pressure assessment, it is possible to obtain a foot pressure distribution map, which plays an important role on the monitoring of foot ulcerations (of particular importance for diabetes patients). In addition, foot pressure maps enable measurements of foot-function indexes such as arch index, which provide the evaluation of the arch type of each individual that is also related to injuries in runners [60].

Furthermore, the dynamic evaluation of the foot plantar pressure can also aid clinicians on the gait-related pathologies diagnosis [61]. The gait cycle is divided into two main phases: stance and swing, which present many subdivisions [62]. The subdivisions of the stance phase can be detected by the plantar pressure variation and it is critical for the control of wearable devices for gait assistance [54,63].

Additionally, the measurement and analysis of joint angles can provide benefits for clinicians and therapists, since it is used on the evaluation and quantification of surgical interventions and rehabilitation exercises [64]. Moreover, such measurements can be applied for training athletes [65] and the kinematic data have been employed on the control of neural prostheses [66]. Furthermore, wearable sensors can be used on healthcare applications [23]. To that extent, significant advances in sensor technology, wireless communications, and data analysis have enabled a change of scenario, where the health condition assessment is not limited to clinical environments [67]. Thus, it is also possible to monitor different physiological parameters for patients at home, which is especially desirable for the elderly population and people with locomotor disabilities [21]. Among many important physiological parameters, abnormalities on the heart rate (HR) and breathing rate (BR) are important indicators of some cardiovascular diseases [68], fatigue [69], apnea [69], and respiratory abnormalities [70].

For physiological parameters monitoring, several electronic sensors based on different approaches such as piezo-electric films, dry textile electrodes, and flexible capacitive electrodes (among others) have been proposed throughout the years. Such technologies are summarized in some published review works [21,23]. However, in general, as electronic sensors are sensitive to electromagnetic interferences, it inhibits their application in magnetic resonance imaging (MRI) [71] and can also present inaccuracies related to the electromagnetic field produced on the constant activation of electric motors in wearable robots.

For the plantar pressure and ground reaction forces (GRFs) monitoring there are three major techniques for foot plantar pressure assessment: imaging technologies [72], force/pressure distribution platforms [73], and instrumented insoles [74]. Imaging technologies generally employ expensive equipment and complex signal processing [59]. In addition, these issues scale when the analysis is made in computed tomography machines, which, besides the high cost, inhibits the dynamic analysis of the plantar pressure [72].

As a more affordable option with the possibility of performing dynamic analysis, force platforms are used on the plantar pressure assessment. These platforms generally have a matrix of pressure sensing elements arranged in a rigid and flat platform [59]. Even though they provide measurements of the foot plantar pressure and 3D dynamics, they also lack in portability, restricting the tests to laboratory or clinical environments, where there is a limitation on the number of steps per trial. This drawback inhibits the application on wearable robotics, remote and home health monitoring, which is a trend in healthcare applications with the advances in wireless sensor and communication technologies [75]. Another drawback of force platforms is the so-called foot targeting effect, where the users alter their natural gait pattern in order to correctly place the foot on the platform, which leads to inaccuracies on the analysis [76]. Consequently, it leads to the necessity of hiding the platform on the ground and repeating the test until a natural gait pattern is obtained with the foot placed within the platform boundaries [73].

Instrumented insoles became a feasible option to the force platforms with the possibility of being used inside a shoe, thereby resulting in a portable device to be assessed outside the laboratory environment, for remote health monitoring and wearable robotics applications [59]. In addition, another advantage of instrumented insoles is the possibility of monitoring the plantar pressure during daily activities with the natural gait pattern of the users. However, mainly due to the sensor technology employed (generally electronic sensors), such insoles can present instability on the measurement (with false positives and false negatives) and a lack of resistance to the impact loads that commonly occurs in the gait cycle [61]. Another issue of instrumented insoles is the number of sensors, which resulted in a low spatial resolution for the plantar pressure analysis [59]. Studies regarding weight distribution along the human foot indicate the need to assess 15 pressure areas that support most of the body weight as discussed in reference [74]. Thus, a sensor system for monitoring plantar pressure may need 15 sensors positioned on each of those 15 critical points.

However, previous works generally aim to a system simplification and the number of sensors is generally 5 to 7 sensors distributed on the foot [59]. Even though a higher number of sensors can be achieved with sensors based on smart textiles and custom fabrics, they generally present performance limitations such as low repeatability, hysteresis, creep, and nonlinearities [59].

The drawbacks and limitations of the conventional electromechanic sensors for monitoring of the previously mentioned parameters can generally be overcome by POF sensor technologies, which present biocompatibility and compactness desirable for applications that involve human contact. In addition, the high flexibility of POFs enable their positioning on the most complex human joints for measuring physical parameters with a large dynamic range. The multiplexing capabilities in conjunction with the possibility of developing a low-cost system open new possibilities on the development of cloud-enabled healthcare systems and smart systems such as smart homes and smart textiles, which will be addressed in Section 4.

**Figure 3:**
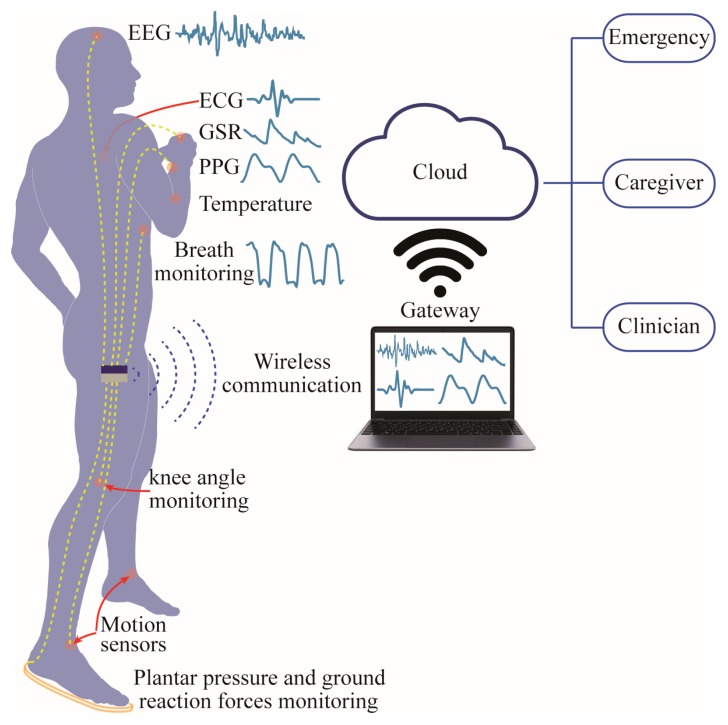
Schematic representation of a multisensory architecture for remote health monitoring.

All the aforementioned sensors can be integrated in architectures for remote health monitoring, where all the data acquired by the sensors can be transmitted from a personal or home gateway to a remote database or to the cloud using a secured connection [67]. In addition, all the data can be processed and then transmitted to the healthcare provider [21]. For this reason, issues related to interoperability and integration have been studied, as summarized in reference [77]. However, it is important to note that these issues can be reduced if the multiplexing capabilities of optical fiber sensors are considered. In this case, all the information will be transmitted using a single optical fiber cable, which not only reduces the issues regarding integration and interoperability when a system with different sensors is used, but also reduces the size of the system as well as the number of components for data acquisition and transmission. Aiming at this background, eHealth architectures using optical fiber sensors have already been proposed [78]. Figure 3 presents an overview of the architecture using conventional sensors for health assessment with the data transmitted to the cloud. In this example, the sensors include electroencephalogram (EEG) for the assessment of the cerebral signs, electrocardiogram (ECG) for assessment of the cardiac conditions, sensors for galvanic skin response (GSR), as well as conventional sensors for temperature, angle, motion, plantar pressure, and breathing monitoring.

## 3. Polymer Optical Fiber Sensors: An Overview

### 3.1. Gratings Incription and Polymer Optical Fiber Materials

The application of optical fibers as sensors has been studied throughout the years and there are many sensing approaches using optical fibers such as interferometers [79], nonlinear effects [80], surface Plasmon resonance [81], fiber Bragg gratings (FBGs) (uniform [82] and non-uniform [83]), and intensity variation-based sensors [84]. For simplicity, the type of sensors can be divided as sensors based on intensity (or power) variation on the fiber and wavelength-based sensors. Intensity variation-based sensors can be employed to evaluate the beam dispersion and phase deviation for measurement as in a turbidity sensor [84]. The differences in light coupling between two fibers are also used for sensing parameters such as strain [56], curvature [85], and acceleration [86]. However, most of the POF intensity variation-based sensors take advantage of the polymer flexibility to measure the transmission losses on a bending fiber, where this principle is used in conjunction with the other effects on the fiber such as the stress-optic effect to measure not only angles [87], but also force [61], temperature [7], and humidity [88]. Such sensors have the advantageous features of low cost, ease of implementation, and simplicity on the data analysis and acquisition [84].

The other major type of sensor is the wavelength-based sensors, in summary, the measurand is compared with the variations on the wavelength of the sensor region. Such sensors can be accomplished with the production of diffraction gratings [89], interferometers (in their many forms such as Mach–Zehnder [90], Fabry–Perot [91], Sagnac [92], and Michelson [93]), and surface Plasmon resonance [94].

As one of the most common wavelength-based sensor, FBGs are created through a refractive index modulation when the fiber is exposed to a periodic intensity pattern, where a specific wavelength (the Bragg wavelength) is reflected [89]. This is essentially a wavelength-selective mirror. Such a grating structure can be obtained by using lasers to modify the optical fiber, and this can take several forms, such as ultraviolet (UV) laser irradiation through holographic techniques, phase mask [89], or direct-write of the grating pattern in the fiber core [95].

Peng et al. reported the first polymer optical fiber Bragg grating (POFBG) inscribing with 325 nm continuous wave (CW) He-Cd and 248 nm pulsed Kypton Fluoride (KrF) lasers, although a period ablation was reported with the KrF laser [96]. As a result, He-Cd lasers were for many years the preferred option for grating inscription in POFs. At first, POFBGs were inscribed in the 1550 nm region mainly due to equipment availability associated with telecommunications applications [97,98]. However, the majority of POF has higher attenuation in the 1550 nm wavelength region and as a logical consequence grating inscription oriented itself towards lower wavelengths. Considerable progress in low-cost interrogation using complementary metal-oxide semiconductors (CMOS) made inscription in the 850 nm region more attractive [99]. However, due to improvements in polymer photosensitivity characterization and analysis, along with the knowledge that photosensitivity increases for deeper UV wavelengths, KrF lasers, operating at 248 nm, were widely employed in FBG inscription in the last few years as they can result in inscription times as low as some ns for different materials [82] and with a single laser pulse of 15 ns duration for doped POFs [100]. In addition, further developments on the laser pulse duration have led to the fastest inscription time to date, 8 ns, with the Nd:YAG laser [101].

Concurrently with the foregoing developments on UV lasers for grating inscription in POFs, ultrafast lasers (light pulses shorter than tens of picoseconds) have been researched, leading to the popularization of fs lasers as commercial solutions for micromachining in transparent materials [102]. In fs lasers, the FBG inscription occurs mainly by multi-photon absorption, where the carriers are excited by the absorption of photons and the energy transfer occurs from the electron to the lattice due the electron-lattice scattering [103]. This inscription mechanism results in a precise energy deposition, since the interaction between the light and matter occurs only near the focal volume of the laser beam, which results in a selective modification of the material properties, i.e., only a small volume of the material is modified [104]. In addition, the annealing of the lattice that occurs with pulse duration of nanoseconds does not occur in ultrafast laser pulses [104]. Thus, on the fs laser FBG inscription, the thermal diffusion is suppressed as well as melting and re-solidification effects, which can induce additional (and undesired) structural modifications on the material [103].

Aiming at the advantages of fs lasers in micromachining of transparent materials, Theodosiou et al. [95] presented a major breakthrough regarding the FBG inscription in CYTOPs (Cyclic transparent optical polymer) using a direct write, plane-by-plane inscription method with optimized parameters, which enabled the FBG inscription in CYTOP fibers within a few minutes. In addition, the proposed technique also enables the control of the mode excitation by means of limiting the inscription to a predefined part of the fiber core, which results in single peak FBGs, even in multimode fibers as the CYTOP [95]. This advantageous feature also enables the inscription of long FBG arrays in CYTOP fibers [105]. Thus, many techniques for FBG inscription and sensor fabrication in POFs have been proposed, where such techniques can be used for different POF materials. A deeper discussion about the many methods and advances in FBG inscription and its many variants in different POF materials is presented in the following review works: [45,89,106].

Of all POF materials, PMMA (Polymethyl methacrylate) is the most employed material in POF production [107]. However, PMMA has a low glass transition temperature (T_g_) compared to some of the other polymer materials for fiber fabrication, such as polycarbonate (PC), which can limit its application at higher temperatures [108] if the humidity variations are not considered. Furthermore, the higher moisture absorption capability of PMMA can harm its application in temperature or strain sensing where a humidity cross-sensitivity is undesirable [99].

In order to mitigate the issue of humidity cross-sensitivity, POFs made of cyclic olefin copolymer such as TOPAS grade 8007 [99,109] and 5013 [110,111] and cyclic olefin homopolymer such as Zeonex 480R [112] can be employed. Gratings inscribed in fiber made of these materials demonstrated a humidity sensitivity at least 30 times lower than that of PMMA POFBGs. However, the glass transition temperature can vary significantly among different grades of TOPAS. For instance, TOPAS 8007 presents T_g_ of only 78 °C, which is even lower than the PMMA glass transition temperature (110 °C) [109] and which implies that fiber cleaving parameters are quite different for POFs made of PMMA and TOPAS [113]. The low glass transition temperature of TOPAS 8007 fibers severely limits the range of temperature sensing. In order to overcome this, POFs made of another grade of TOPAS with a T_g_ of 134 °C was demonstrated [110,111]. Another polymer material employed for high temperature and strain sensing is Zeonex 480R, which has a T_g_ of 138 °C. Zeonex 480R material presents further advantages compared to TOPAS 5013, such as superior drawability, that allow a more robust fabrication of microstructures in the fiber [112].

Both TOPAS and Zeonex have low loss at THz frequencies and have been applied extensively in this field as well [114,115,116]. In addition to the aforementioned POF materials, PC polymer can also be used for fiber fabrication [117]. PC has a T_g_ of 145 °C, which is higher than TOPAS 5013 and Zeonex 480R [108]. Furthermore, PC POFs can withstand higher stresses when compared with PMMA and TOPAS POFs [108]. For these POFs, the use in the 1550 nm region is limited to a few centimeters and, for this reason, most POFBGs are inscribed in the 850 nm wavelength region [118] with some progress on the 600 nm wavelength region as well [119,120]. In order to tackle the limitations imposed by the higher attenuation of POFs, graded-index CYTOP fibers have been proposed [121]. In CYTOPs, the carbon-fluoride bonds replace carbon-hydrogen in the polymer backbone, resulting in much lower losses in near infrared (especially in 1550 nm) when compared with PMMA POFs [121]. In addition, this material also has low dispersion [121] and these advantages have led to the rapid widespread use of CYTOPs as commercial solutions for POFs. Moreover, the direct connectorization of POFs in optical connectors instead of the butt-coupling in optical fiber cables is also an important progress towards commercialization of POF sensors, as further discussed in reference [45].

The advances in materials processing also opened new avenues for POF development, where novel techniques for fiber fabrication such as the light spinning polymerization process (LPS) enable the development of POFs with customized optical and mechanical properties (called LPS-POFs) [122]. In this case, the POF is created from a mixture of monomers, which can result in a POF with outstanding strain limits of even 200% and Young’s modulus as low as 15 MPa, resulting in sensors with sensitivities some orders of magnitude higher than the ones obtained with the aforementioned POFs [118]. In addition, biocompatible and biodegradable POFs have been recently proposed [123], which represent a major advance towards in vivo monitoring of several parameters, including intracranial pressure [36] and strain in tendons [124] as well as biosensors for physiological parameters monitoring [3].

**Figure 4:**
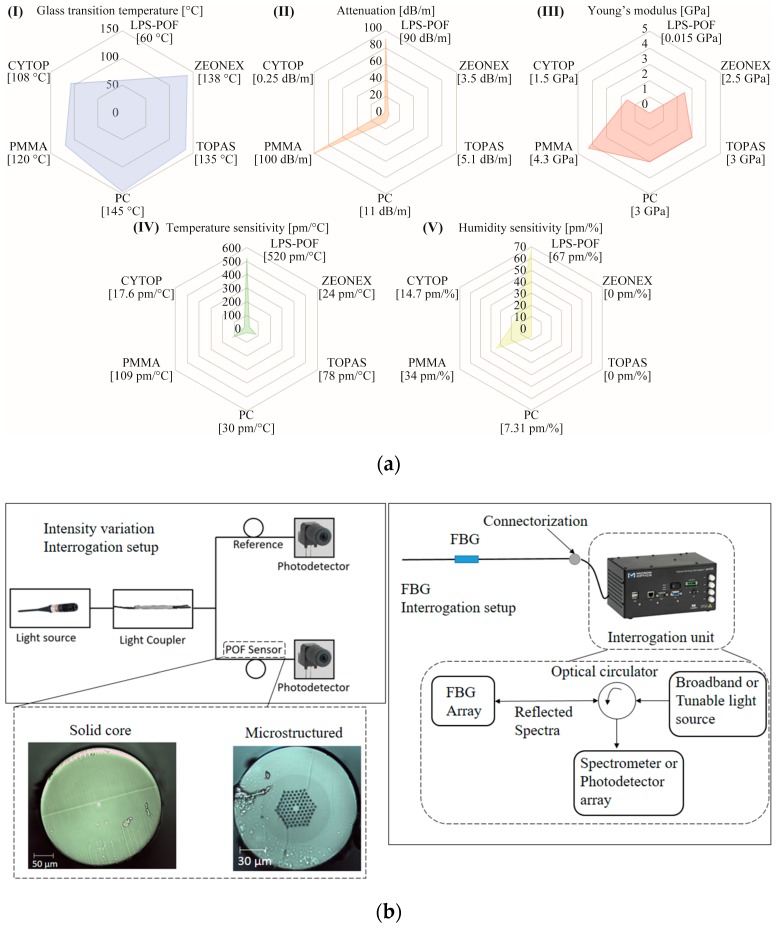
(**a**) Properties of different polymer optical fiber (POF) materials. (**I**) Glass transition temperature, (**II**) optical attenuation, (**III**) Young’s modulus, (**IV**) Temperature sensitivity (considering fiber Bragg gratings (FBGs)) and (**V**) Humidity sensitivity (considering FBGs). (**b**) Interrogation setup for intensity variation-based and FBG sensors. Figure inset shows microscopic images of POFs.

It is also worth mentioning that POFs also have higher flexibility in fabrication due to the lower temperatures and pressures involved in their fabrication. Thus, low-cost methods, such as polymer recycling depicted in reference [125] and 3D-printing methods [126,127] have been proposed for the production of POFs. In order to summarize the functionalities of the different POF materials discussed and to provide some guidelines for the materials choice, Figure 4a presents an application map of the different POFs discussed, where the POFs are categorized with their temperature, strain, and humidity sensitivities as well as their attenuation and Young’s modulus. It is possible to observe in Figure 4a that LPS-POFs have higher sensitivity than the other materials for temperature and humidity. In addition, the low Young’s modulus of such fibers also enables the development of force sensors with higher sensitivity than the other fibers. However, their optical attenuation is also higher than the other POFs as well as their low glass transition temperature that limits the highest temperature that the fiber can operate. Thus, Figure 4a shows guidelines for the POF material selection that can be performed according to the desired application. The interrogation setup for the intensity variation-based sensors and FBG sensors are presented in Figure 4b. In addition, the microscopic images of some POFs (solid core and microstructured) are also shown in Figure 4b. For intensity variation-based, the setup needs only a light source and a photodetector (or power meter) in order to measure the power variation as a function of a predefined parameter. The setup for intensity variation sensors is commonly used in transmission mode (as shown in Figure 4b). Moreover, there is a possibility of using another fiber in order to verify the light source power deviations, which is a source of errors in this sensing technique. On the other hand, FBG sensors do not present sensitivity to light source power deviation, since the measured data is wavelength-encoded. Thus, the interrogation setup for these sensors need a FBG interrogation unit that generally comprises of a broadband light source, an optical circulator, and a spectrometer (or photodetector arrays). In FBGs, changes in the period of the gratings lead to shifts in the Bragg wavelength, which is correlated with the parameter to be measured. Although FBGs are intrinsically sensitive to strain and temperature (due to the thermal expansion, photoelastic, and thermo-optic effects), they can be embedded or coated with different materials or can be positioned in mechanical systems in order to measure different physical or chemical parameters.

### 3.2. Viscoelasticity in Polymer Optical Fibers

It is noteworthy that the POF material features are two-fold. On the one hand, the lower Young’s modulus of POFs and higher strain limits enable developing sensors with higher dynamic range and much higher sensitivity when compared with the ones in silica fibers [118]. On the other hand, polymers are viscoelastic materials, which do not present a constant relation with stress or strain [17]. Furthermore, viscoelastic materials present a hysteretic response between stress and strain [18] and this may be a source of hysteresis and nonlinearities in POF sensors [128]. In addition, viscoelastic materials have a variation of their Young’s modulus in response to different parameters such as strain cycle frequency, temperature, and relative humidity [129]. Therefore, it is necessary to understand and characterize the POF viscoelastic response to propose a compensation of the viscoelastic effects and obtain a more reliable measurement of any POF sensor based on direct stress or strain on the fiber (such as curvature sensor [130], strain [131], and force [61] sensors) prior to their applications in healthcare devices and movement analysis.

**Figure 5:**
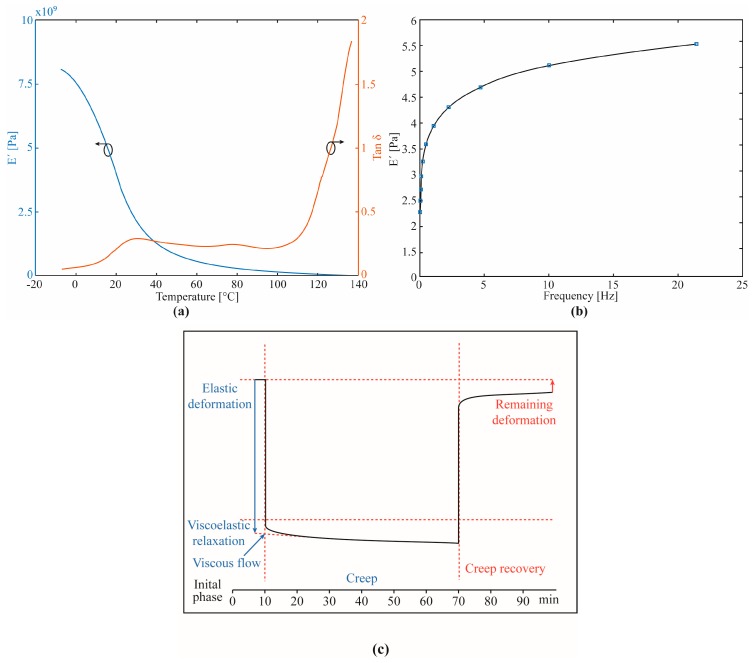
Typical response curves from POFs. (**a**) Temperature response with storage modulus (E’) and loss factor (tanδ), (**b**) Frequency response, and (**c**) Creep response indicating the viscous and elastic part of the polymer response.

In order to characterize the mechanical properties and polymers viscoelastic response, dynamic characterization tests are performed. The dynamic characterization tests are widely employed for polymers in biological applications [132], automotive [133], aircraft [134], and industrial [135], among others. Dynamic tests on polymers can be made by means of nano-indentation techniques [136] and dynamic mechanical analysis (DMA) [137]. Nano-indentation techniques are more suitable for the evaluation of thin films and microstructures. Moreover, it is also applicable to the assessment of local properties of the polymer [136]. Therefore, the DMA technique is a suitable technique due to the fiber dimensions on the order of millimeters. DMA applies an oscillatory load on the polymer with specific temperature and frequency ranges, which is used to evaluate the polymer glass transition, storage modulus, loss modulus, and stress relaxation [137]. Each of the aforementioned POFs can be characterized using this method, which show the differences in the responses of each polymer material as well as in their different microstructures [128,138,139]. Typically, the polymers present responses to oscillatory loads in DMA similar to the ones depicted in Figure 5 for temperature, frequency, and creep responses (obtained in PMMA analysis [128]) and these responses with respect to each parameter can be used to compensate or eliminate some unwanted behaviors of the POF sensors such as hysteresis, creep responses, and temperature cross-sensitivity.

The knowledge of the POF material features enables the development and performance enhancement of the presented different POF sensors. With this background, it is possible to not only propose novel POF-based sensors for microclimate and torque sensors using the stress-optic effect, but also the performance enhancement of such sensors. Using the concepts of polymer viscoelasticity, different compensation techniques for the POF sensors nonlinearities, hysteresis, and creep response were proposed and validated, where the compensation of sensor hysteresis can be accomplished with the sensor characterization at different angular velocities [140]. In addition, the modelling of the viscoelastic response of POFs can also lead to the reduction of nonlinearities (in conjunction with the hysteresis) [128] as well as the compensation of the polymer creep response [141]. It is also important that the POF sensors can present cross-sensitivity with temperature and frequency due to its material properties dependency with these parameters.

However, the background provided by the fiber characterization leads compensation techniques for such effects as demonstrated in reference [142] for the compensation of temperature cross-sensitivity on the transverse force sensing using FBGs inscribed in CYTOP fibers. Similarly, the compensation of frequency effects on the dynamic measurement of bending angles was proposed for CYTOP fibers in reference [143]. All these techniques are used in conjunction with the thermal treatments on the fiber, where such thermal treatments like the annealing lead not only to the increase on the sensor performance as demonstrated in references [144,145,146] for temperature, strain, transverse force, and humidity, but also increase their drawability and the stabilization of FBGs [147,148,149], since the influence of the annealing on the material properties was also investigated [150]. Therefore, the many developments on the POF materials in conjunction with the evolution on the sensors production and materials characterization and processing have led to the development of high-performance POF sensors readily available for many healthcare applications (discussed in Section 4).

## 4. Polymer Optical Fiber Sensors: Applications in Healthcare

After knowing the current scenario and trends in healthcare as well as the developments on the POF sensing technology, the healthcare applications of such sensor system are depicted in this section. The applications are divided into five different categories, as shown in Figure 2, where the applications are the instrumentation of wearable robots (exoskeletons, prosthesis, and orthosis) with different approaches of POF sensors. Then, we discuss the instrumentation of other healthcare devices using POF sensors. These devices are a SW and a system for gait assistance with functional electrical stimulation (FES). Such devices are either not robotics (such as the FES-assisted gait) or not wearable (such as the SWs). Thereafter, the applications on movement analysis are depicted, where many wearable sensors using POFs are presented and discussed. As another approach for human movement assessment, instrumented insoles and intelligent carpets are discussed as a feasible approach to detect plantar pressure during the gait or other activities. Finally, the discussion of physiological parameters monitoring using POF sensors is presented. Even though many of the systems for this purpose are not wearable, it is important to discuss their many applications and possibilities for further improvement in order to obtain wearable systems for the assessment of many physiological parameters.

### 4.1. Wearable Robots Instrumentation

**Figure 6:**
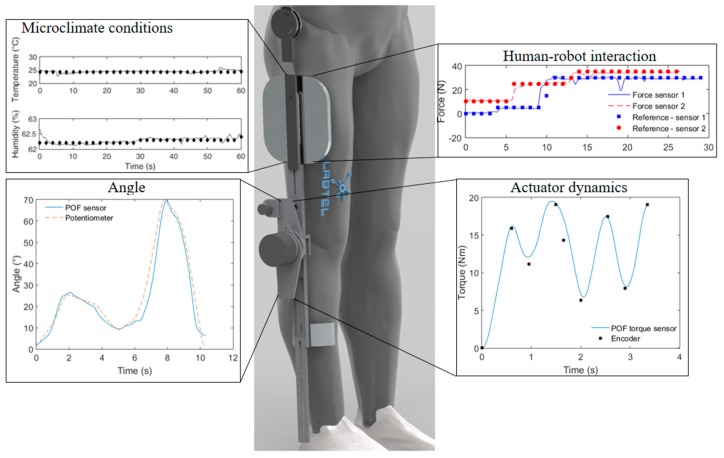
POF-based sensors on wearable robots’ applications.

A POF sensor system for angle measurement on the knee joint of a robotic lower limb exoskeleton was proposed in reference [151]. The exoskeleton is positioned on a chair and is used to provide assistance on knee flexion and extension movements with predefined angular velocities and resistive (or assistive) torques. The sensor system comprises of a POF made of PMMA with a lateral section produced following the guidelines depicted in reference [5]. The lateral section is made by abrasive removal of material in order to remove the fiber cladding and part of this core in a specific region, where this lateral section increases the sensitivity and linearity of the sensor for angle measurement. As discussed in Section 3, POF sensors may present hysteresis due to the material properties, which, in this case, is compensated using a technique depicted in reference [140], where the sensor is characterized at different angular velocities in order to obtain an equation that is able to compensate the hysteresis for each angular velocity. After the sensor production, the system is positioned on the robotic device (as summarized in Figure 6) and flexion/extension movements are performed at different angular velocities. The POF curvature sensor response is compared with the potentiometer already positioned on the device and good agreement was obtained between the sensors. Such a result indicates the feasibility of using a low-cost POF sensor system to measure angle in an exoskeleton joint with additional advantages of electromagnetic field immunity and a more compact structure. As many wearable robots are intended to assist the gait of the user, the evaluation of the POF sensors in gait movement using a lower limb exoskeleton was also performed [54]. Once again, the exoskeleton’s potentiometer was compared with the proposed POF sensor system (based on intensity variation in a POF with a lateral section) and good correlation was found between both systems with deviations as low as 3° considering the whole test.

Similarly, POF sensors based on intensity variation were also tested in different exoskeletons in order to evaluate their repeatability and interchangeability. Thus, in reference [54], the POF sensors are positioned on a modular exoskeleton for gait assistance [152]. The system was evaluated in flexion/extension cycles for the knee joint as well as in gait tests, where the proposed sensor system was compared with an encoder already positioned on the device. In this case, the errors were below 5° when both systems are compared (POF sensor and encoder). The positive feedback of the joint angle measurements in wearable devices in conjunction with novel multiplexing techniques for intensity variation-based POF sensors [153] indicate the possibility of obtaining in the near future a bilateral lower limb exoskeleton instrumented with a single POF cable with one curvature sensor (based on intensity variation) for each joint. It is also worth noting that a similar approach can also be made with FBGs in POFs, since their functionality and cross-sensitivities compensation were also investigated in reference [143]. Thus, we can envisage novel trends on the POF angle instrumentation in wearable robots, where different sensor approaches can be used. In addition, due to their higher flexibility and strain limits in conjunction with the good performance already demonstrated, we can expect that the POF sensors will be a viable (or even vital) technology for the angle instrumentation in the next generation of soft robotics.

As an important parameter on the exoskeleton control, the human–robot interaction force assessment was also investigated using POFs. It is important to evaluate the force (or torque) that the user applies on the robot in order to evaluate its development on a rehabilitation treatment or as a feedback for impedance control of wearable robots in order to minimize the force that the user applies on the robot [22]. In both scenarios, the POF sensors were tested and validated as a suitable option for the instrumentation. In reference [56], an intensity variation-based POF sensor system using the light coupling principle was proposed and validated in a lower limb exoskeleton with impedance control with 10 levels of assistance (or resistance) of the knee flexion/extension movement. In this case, the proposed POF sensor is compared with the strain gauge for each of the assistance levels on the impedance controller. The relative deviation between the devices was lower than 8%. However, it is worth noting that the proposed POF sensor can be positioned directly on the shank support of the wearable robot, which results in the direct measurement of the torque in the point that it is applied (see Figure 6). On the other hand, the electronic strain gauge used for comparison is positioned on the exoskeleton structure, which may not provide a direct measurement of the applied force, as it acquires the correlation of the applied force with the strain on the robotic metallic structure.

The human–robot interaction force was also assed during the gait in reference [154]. In this case, by taking advantage of the POF flexibility and compactness, a novel 3D-printed POF-embedded instrumented shank support for exoskeletons was proposed. The support is made with the 3D-printing performed directly on the fiber, where the proposed system showed not only the capability of measuring human–robot interaction forces in different protocols (seated flexion/extension cycles and gait cycles), but also in different devices, where the proposed 3D-printed support was positioned on a lower limb exoskeleton and on a passive orthosis for knee stabilization during the gait. Furthermore, since the proposed shank support is in direct contact with the user’s legs, additional POF sensors were added in order to measure the human–robot interaction forces and the temperature and humidity conditions in the interface between the device and user’s legs. Thus, the system proposed in reference [154] provides a direct multiparameter measurement of the forces and microclimate conditions of the user and can be employed in different devices.

In human–robot interaction forces, the higher precision and multiplexing capabilities of FBGs can provide advantageous features on the multipoint (or quasi-distributed) assessment of such parameter. Aiming at these advantages, a multipoint system for human–robot interaction force assessment using FBGs inscribed in CYTOP fibers was proposed in reference [155]. Once again, the fibers are embedded in a 3D-printed structure. However, in this specific case, the FBGs are embedded in two shank supports, one anterior and one posterior, in order to obtain the force distribution in two distinct points of the leg on the knee flexion/extension cycles assisted by an exoskeleton. Despite the many advantages of POF sensors, the FBGs need an interrogation unit that, generally, is a high-cost solution with low portability and intensity variation-based sensors suffer from the sensitivity to light source power deviations. Thus, as the path for wearable robot’s instrumentation using POFs is fruitful there is still room for improvement on the sensor systems and interrogation techniques.

If a wearable robot is concerned, one of its main components is the actuator, which is responsible for delivering the mechanical power for moving the user’s joint or assisting the movement. As an important actuator technology, series elastic actuators (SEAs) are a core technology in wearable and assistive robotics due to their compliance and relative simplicity on the design and control [156]. The actuator comprises of an electric motor in series with a spring (or elastic element), which is in contact with the human joint, making it suitable for the development of compliant active orthosis [49]. The instrumentation of a SEA was proposed in reference [157] using intensity variation-based POF sensors to measure the spring deflection as well as the torque on the actuator output (used on the control of the robotic device). A novel technique based on the stress-optic effect on the measurement of the actuator torque was proposed, which leads to the reduction of components of the actuator instrumentation that initially were two encoders and can be reduced to only one POF sensor. As an important parameter on the disturbance rejection on the actuator as well as a means of increasing the accuracy on the actuator torque measurement (which has a direct impact on its control accuracy and robustness), a quasi-distributed sensor system for measuring multipoint spring deflection was proposed in reference [158]. The system has eight FBGs inscribed in a CYTOP distributed in different points of the SEA’s spring, where it was demonstrated that the deflection measurement in those points can not only lead to the rejection of external mechanical disturbance on the actuator, but also the increase of the accuracy on the torque estimation by using sensor integration techniques such as the Kalman filter [159]. In this case, the use of the Kalman filter has led to a seven-fold increase on the torque measurement accuracy.

As the first application of POF sensors in soft robotics, Casas et al. [160] proposed a POF sensor based on light coupling between fibers for the instrumentation of a wearable robot for gait assistance using artificial tendons in the actuator unit. The proposed wearable robot is positioned on the user’s ankle and provides assistance on this joint movement during the gait by using electric motors in series with the artificial tendons (tendon-driven actuator). In this case, the POF sensors are positioned on the tendons in order to assess the strain on these components. The use of POFs in this application can result in a higher degree of control of this actuator, since the deflection on the tendon was not measured before the application of the proposed POF sensor system and since none of the conventional sensors were able to withstand the high strain on the tendons and do not comply with the requirements of high flexibility and low Young’s modulus.

An overview of the previously presented applications of POF sensors in wearable robotics is presented in Figure 6, where the discussed sensors as well as their positioning and responses are depicted.

### 4.2. Polymer Optical Fiber (POF) Sensors on the Instrumentation of Healthcare Devices

Besides the wearable robots, there are some devices commonly applied in locomotion assistance. As the first discussed application is such devices, a POF-instrumented insole was applied in a FES system for gait assistance in [54]. In this case, bilateral FES-induced quadriceps and hamstrings contractions are used to emulate knee function during gait, which was applied using adhesive 5 × 10 cm electrodes whose activation and tuning are based on a finite state machine. The finite state machine sends the activation commands based on the gait phase of the user, which was originally estimated by means of the joint angles acquired by an IMU. However, the interference on the sensors by the electrodes activation as well as the necessity of frequent calibration of IMUs impose important limitations on the sensor system. In order to tackle these limitations, a POF-instrumented insole for GRFs and gait phase estimation was proposed and validated [61]. The user using the insole inside his/her shoes walks with the gait assisted by the FES while the ground reaction force during the gait is acquired by the proposed insole. Since the gait phases can be estimated by the GRF curve [161], the proposed sensor system was able to estimate the stance and swing phases as well as the stance subdivisions discussed in Section 2, i.e., heel strike, maximum weight acceptance, flat foot, heel off, and toe off. Thus, the gait phase detection system is used to feed the gait phase information for the finite state machine that controls the electrode activation on the FES-assisted gait.

As a common approach for the locomotion assistance, walkers have been used in their many configurations and variants throughout the years. The evolution of control and robotic systems has enabled the development of SWs, which present actuation and a sensor system in order to provide a better assistance to their users [51]. As the first POF sensor approach for the instrumentation of such devices, a system to assess the user health condition while using the SW was proposed in reference [162]. In this case, the sensors are not only positioned on the SW, but also on its user. The sensor system comprised of wearable sensors on the user for measuring heart and breath rates as well as a POF sensor system on the device’s handles in order to assess the pulse oximetry (important indicator of the user fatigue and health condition). Such an approach enables novel control strategies for the healthcare device in which the health condition of the user is also considered, instead of just the SW condition or the ambient positioning and tracking of the device.

In addition, in order to obtain a multiparameter sensor system for SW instrumentation, a quasi-distributed sensor system using POFBGs was proposed in reference [163], where the FBGs were positioned on the walker’s structure to provide assessment of the device’s structural health and human–robot interaction. With the proposed sensor system, it was possible to track the user movement intention when the user performed basic commands such as go forward, stop, turn left, and turn right. In addition, the responses of the FBGs were analyzed in the frequency domain for tests in different floor conditions. In this case, the roughness of the floor surface was changed and the FBGs were able to track such differences by measuring the vibration of the walker’s structure, which can be a feasible approach on the localization and mapping of the mobile robot in a structured environment, i.e., an environment where the positions and floor roughness are known a priori.

**Figure 7:**
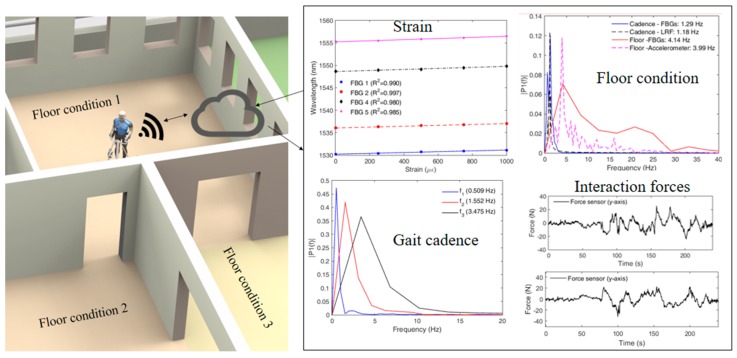
POF sensors on the instrumentation of smart walkers (SWs).

In order to summarize the applications discussed in this section, Figure 7 shows an overview of POF sensors applications in healthcare devices (especially the SW), where it can be seen that there is much room for the development of novel control strategies for these devices. Moreover, with the developments in data transmission and IoT (Internet of Things) (discussed in Section 2), one can envisage the application of such sensors in architectures for cloud robotics and remote health monitoring.

### 4.3. POF Sensors on Human Movement Assessment

Unlike the applications in wearable robots depicted in Section 4.1, where the sensors are positioned on the robot structure, in human movement assessment, the sensors are positioned on the subject. This small difference brings many instrumentation challenges, since the human body has variations on the center of rotation of many joints, more degrees of freedom for each movement, and higher joint misalignments when compared with the structures of wearable robots. In this case, the sensors need to be more robust to misalignments and the constraints of size and weight are even higher for movement analysis applications. On the hand, sensors such as interaction forces and actuator dynamics are not needed in this application field, indicating fundamental differences between the wearable robots’ instrumentation and the application of POF sensors in movement analysis.

There are many applications of POF based on intensity variation as wearable solutions for joint angles assessment. POFs are preferred in rehabilitation purposes due to its biocompatibility and are more rugged and safer than the silica optical fibers [11]. Lee et al. [164] proposed an optical fiber angular sensor to measure the human joint angle based on macrobending power loss. The phototransistor voltage and angle correlation with different fiber tip angles (0°, 20°, and 35°) were investigated. Results shows an asymmetric dependence of voltage and angle for 20° and 35° tip angles.

Dunne et al. [165] developed a wearable garment-integrated curvature sensor for monitoring the seated spinal posture and the subject posture is evaluated by a two-leaved decision tree model for posture classification. The posture classification made by a decision tree algorithm is compared with the classification from a visual analysis of professionals. However, the lack of agreement between the eight experts presents a problem for system evaluation and for long-term monitoring of seated posture. Williams et al. [166] also investigated the spinal curvature. Such research is limited to lumbar curvature. Results of the lumbar monitoring were compared to a video-based method and good precision was proven. Donno et al. [167] developed a POF goniometer capable of measure higher angles (up to 90°). Dynamical tests are conducted in three frequencies: 0.1 Hz, 1.25 Hz, and 5 Hz with good accuracy between and low hysteresis of the POF sensor and the reference potentiometer. The proposed goniometer can be applied for knee joint angle measurement by athletes to test and train their exercises and to physiotherapy rehabilitation. Bilro et al. [168] presented a similar approach to knee angle monitoring. However, the sensor is characterized in quasi-static tests and the sensor is evaluated with a video-based system, which accounts for only the movements on the sagittal plane. The wireless prototype of the POF sensor was also presented. Moreover, a characterization of the same POF sensor principle was made to apply the POF goniometer to elbow angle measurement. Similarly, a knee angle measurement system on the sagittal plane was proposed in reference [169], where different approaches for the lateral section were investigated. In order to propose a reliable portable sensor system for joint angles monitoring, an analysis of thermal treatments on the POF in conjunction with the application of compensation techniques based on material features for a portable POF-based sensor system with wireless communication was proposed [170]. In this work, configurations of the curvature regions and guidelines for the fiber length and mounting were investigated and validated in knee and elbow joint angle measurement.

The advances on material processing with the development of novel POFs have pushed the boundaries on POF applications on human movement analysis. Guo et al. [171] presented a stretchable FBG-based optical (SFO) strain sensor for wearable and skin-mountable detection of human activities, interrogated by a compact dual-comb, mode-locked fiber laser in free running. The stretchable sensor is fabricated by embedding sinuous-shaped FBG at an off-center position of a stretchable polydimethylsiloxane (PDMS) substrate. The proposed SFO sensor can also be used to detect bending and torsion deformations with capability of directional discrimination. For measurements of the strain-induced shift of the Bragg wavelengths, a novel free-running mode-locked fiber laser with coherent dual-comb output is built to interrogate the FBG based on dual-comb spectroscopy (DCS), enabling fast-speed spectral detection of multiplexed sensor array with a single photodiode (PD). To investigate the sensor response upon strain deformations, finite element modeling (FEM) was employed. The SFO sensor can be assembled on clothing or directly mounted on human skin to monitor diverse human activities from breathing, phonation, and facial muscle movements, to marching, jumping, squatting, bending, and torsion of the joints.

As another development of material processing on the production of novel POFs for movement analysis, Guo et al. [172] presented the design and fabrication of a highly stretchable optical strain sensor based on dye-doped PDMS optical fibers. PDMS is thermally stable, isotropic, homogeneous, and highly transparent in a wide spectral range [173,174], the flexibility and stretchability of the PDMS fiber make it especially attractive for the sensing of large strains. Dye-doping to the PDMS can be achieved by adding dye molecules to the PDMS precursor prior to curing [175]. The dye molecules induce wavelength-dependent absorption properties to the doped PDMS fiber, and tensile strain was measured by monitoring absorption changes based on absorption spectroscopy. Since the proposed sensor is highly flexible, stretchable, and sensitive, it was mounted onto a rubber glove using epoxy to detect the finger motions and was tested for monitoring muscle motions by attaching the sensor to a volunteer’s neck, being capable of sensing the voice vibrations generated by speaking and the strains associated with inhaling and exhaling, with good repeatability and responsiveness.

In fact, the advantageous features of POF sensors can be used in conjunction with other conventional sensing technologies in order to provide complementary approaches for increasing the sensor accuracy or to provide additional information of the system. Considering this background, a sensor system comprised of IMUs and POF intensity variation-based curvature sensors was proposed in [176]. The system used the IMUs capability of measuring angles at three dimensions with the high accuracy of POF curvature sensors for single plane measurements, where the complementary information was combined using the Kalman filter. The proposed sensor system was evaluated in human joint angle assessment and can be regarded as a low-cost solution with high reliability and possibility of multi plane and multi joint angle assessment. Following a similar approach, the use of POF curvature sensors in conjunction with markerless camera-based systems for joint angle assessment was proposed in reference [177]. In this case, a correlation was observed between the lengths of the arm with the errors presented by the camera-based system on elbow angles estimation. Such correlation was only possible to be acquired using a wearable sensor technology (such as the POF curvature sensors), which resulted in an integrated system for angle assessment using POF sensors and markerless camera-based system.

**Figure 8:**
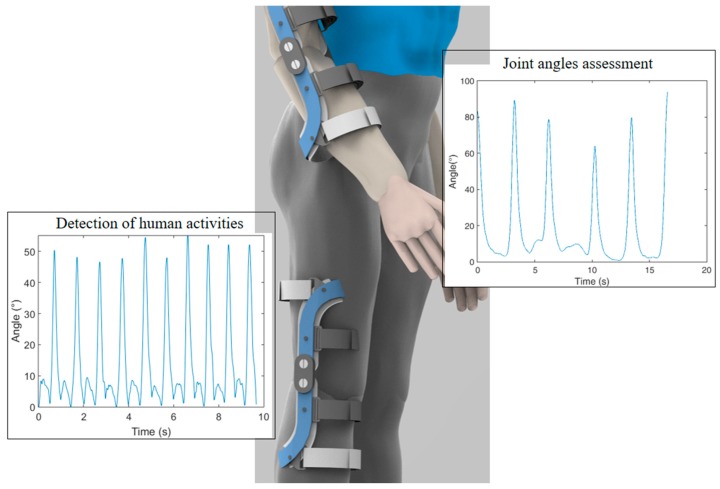
POF sensors applications on human movement.

Figure 8 summarizes the healthcare applications discussed, where the applications of POF sensors on human movement were thoroughly discussed. However, it is worth noting that new machine learning approaches for human activities detection can also be applied in conjunction with these sensors systems in order to obtain intelligent instrumentation systems for online extraction of characteristics of each activity as well as remote health monitoring [21].

### 4.4. Plantar Pressure Assessment Systems Using POF Sensor Technologies

Plantar pressure measurement systems were initially proposed with silica FBGs due to their wider availability and multiplexing characteristics [41,178]. Similarly, sensing pads for foot pressure monitoring were also proposed using FBGs in silica fibers [179]. Such systems are able not only to quantify the GRF and plantar pressure during the gait, but also for the analysis of pronation/supination of the foot. By taking advantage of the higher strain limits and pressure sensitivity of POFs, Vilarinho et al. [10] proposed a cork insole embedded with POFs with a 5-FBGs array. The proposed POF sensor system showed higher pressure sensitivity than the one made of silica fibers, which leads to the possibility of higher measurement resolution (considering the same FBG interrogation equipment) and with the possibility of measuring a larger range of users due to POFs higher strain limits.

In an attempt at using a lower cost system, the embedment of POFs in fabrics to obtain an intensity variation-based sensors matrix was investigated [180]. To that extent, Reyes et al. [181] employed an “intelligent carpet” prototype consisting of 116 POF sensor elements to measure gait parameters based on intensity variation principle and developed machine learning models to distinguish and classify different manners of walking. Time series were acquired in pilot experiments capturing several gait cycles on the footprint imaging sensor and they used a set of 14 supervised linear, non-linear, ensemble, and deep machine learning models. They aimed to achieve reliable classification scores for 10 different pre-defined manners of walking of a single user to provide unique gait patterns resulting in variations in the frequency content and amplitude of the temporal signal.

**Figure 9:**
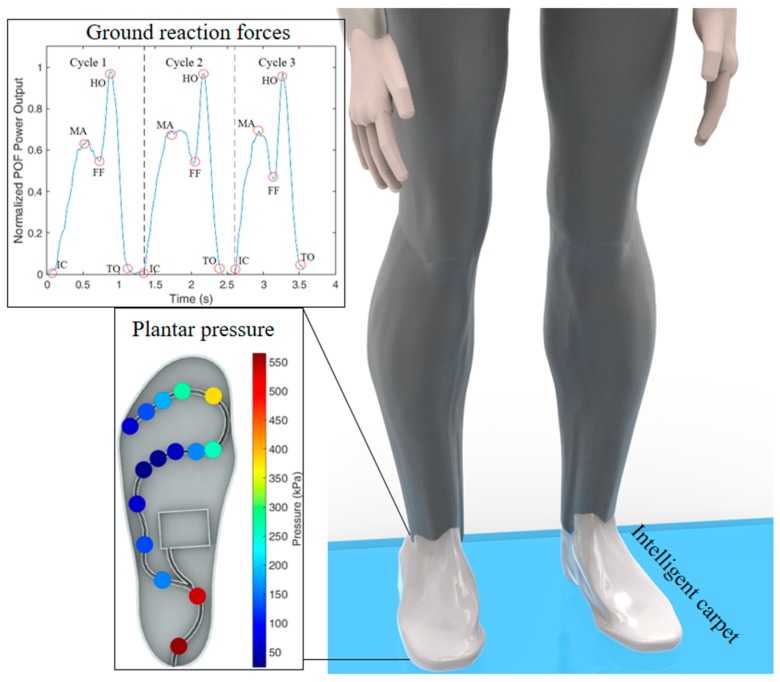
Plantar pressure instrumentation systems using POF sensors.

However, a high number of intensity variation-based sensing elements lead to the necessity of a higher number of photodetector and sources for the sensor system, which can not only reduce the system compactness, but also increase its cost. Thus, conventionally, there is a tradeoff between the number of sensor elements and the cost as well as portability of the system. This paradigm begins to change when a multiplexing technique for intensity variation-based sensors was proposed. In this case, the fiber has a lateral section and light source is side-coupled on the lateral section [153] with two photodetectors, one at each end facet of the POF in order to compensate for light source power deviations. Using this approach, an instrumented insole was proposed with 15 sensing points using POF intensity variation-based sensors in [182], where only two photodetectors were used to monitor the whole system. In addition, the insole was fabricated by 3D printing and the POF was embedded in the 3D-printed structure, resulting in a flexible, low-cost, and highly customizable sensing system. It is also worth noting that the insole uses wireless communication for the data transmission, which leads to a portable system as well. The proposed 3D-printed insole was used validated in a force platform, which shows the capability of the proposed sensing system of measuring the GRF and the center of pressure of the users even when they performed oscillatory movements. Then, the POF-embedded insole was used to assess the plantar pressure distribution during the gait of 20 volunteers, where the sensor system was able to track the plantar pressure at each sensing point and was also able to obtain the GRF curve, which can be used to identify the gait phases. In order to summarize the developments on POF sensors for plantar pressure monitoring, Figure 9 shows an overview of the proposed applications discussed in this subsection.

### 4.5. Physiological Parameters Assessment with POF Sensors

Important applications of POF sensors in healthcare are physiological parameters monitoring where many parameters were investigated throughout the years. As an important indicator of many cardiovascular diseases, Leitão et al. [183] proposed a low-cost POF intensity variation-based sensor. In this sensor, a reflective surface is positioned on the user’s carotid artery’s surface. The sensor is based on the light coupling principle, where the reflected light (the light that returns from the reflective surface) is acquired by a photodetector and the power variation is correlated with the carotid distension. The proposed sensor is compared with a commercial solution for the assessment of carotid distension and good agreement between both sensors was obtained, which indicates the possibility of using the proposed low-cost solution on the assessment of arterial pulse.

The high flexibility of POFs motivates many research groups on embedding such fibers in textiles in order to obtain an integrated sensing solution for vital parameters monitoring [180]. To that extent, a respiration monitoring using POFs integrated in a textile was proposed in reference [13], where the sensor was positioned in different regions of the chest in order to evaluate the type of respiration, e.g., diaphragmatic, upper coastal, or mixed. Besides a new doped POF that enables fast FBG inscription, Bonefacino et al. [184] proposed an FBG inscribed in doped PMMA POF for breathing and HR monitoring. The sensor response presented the shape of a ballistocardiogram curve in the HR analysis and the oscillatory response with the respiratory frequency on the BR analysis. However, in many optical fiber sensor approaches, the sensor response is the sum of the BR and HR. In addition, if the sensor is used under dynamic movements, there will also be a cross-sensitivity on the sensor response due to the movements. In order to tackle this limitation, a POF-based sensor system for BR and HR monitoring under dynamic (or oscillatory) movements was proposed in [185]. The sensor system comprises of a POF-embedded smart textile, where the sensor response is analyzed in the frequency domain and, with filtering in different frequency windows in conjunction with simple classification techniques, the sensor response for BR and HR without influence of dynamic movements is obtained. Furthermore, the integration of the POF-based breathing sensor in a smartphone was investigated in [186], where a portable and compact solution with low cost was obtained and validated.

The integration of POFs in textiles, creating the so-called photonics textiles, was further investigated in reference [187], where the POF was integrated in a textile forming a sensor matrix that was tested under different configurations. The proposed photonics textile was tested on the measurement of oxygen saturation in tissue, where two wavelengths were used in order to deconvolute the impact of oxyhemoglobin, deoxyhemoglobin and obtain the SpO_2_ (percentage of hemoglobin with bound oxygen measured in the pulse). It is worth noting that the analysis of the SpO_2_ signal in the frequency domain results in the HR that can also be used on the assessment of such parameter as demonstrated in references [12,162].

As non-wearable POF sensors technologies that have great importance on the health assessment, some biosensors have been proposed in the last few years. One basic premise of these sensors is to detect a variation (or contamination) on a given sample by analyzing differences on the refractive index of the sample. For this reason, the development of POF-based refractive index sensors is of great importance and many examples can be found in the literature [6,188,189]. The use of POFs as biosensors has been proposed in reference [190], where a PMMA POF was used in a compound parabolic concentrator to measure glucose concentration in different samples by fluorescence variation with a glucose permeable membrane. Another biosensor application was proposed in reference [191], where a PMMA mPOF was used on the selective antibodies detection also using fluorescence in small samples with fluorophore labeled antibodies. In reference [192], the localized antibodies selection is performed using TOPAS POF, which present humidity insensitivity and higher chemical inertia, making it more suitable for this specific application. Recently, pH sensors based on POFs were proposed, where the sensor is based on FBGs inscribed in POFs in different solutions, e.g., hydrogel [193] and aqueous solutions [194]. It is also worth mentioning many advances on photoacoustic effects and ultrasonic detection with POF sensor system that can lead to important advances in healthcare applications [45,195,196]. As a final important application, the thermal profiling assessment radiofrequency treatment was proposed in reference [197] using chirped FBGs inscribed in PMMA POFs, where the sensor high sensitivity enabled the thermal profile detection with millimeters resolution, which is important to assess the thermal ablation on the tissue during radiofrequency treatments.

## 5. Conclusions

This paper presented a thorough review of POF sensors applications in healthcare. The current scenario and trends in healthcare were depicted as well as the new developments on POF sensor technologies, regarding not only the sensors themselves, but also the developments on material processing, analysis, and characterization, which, in conjunction with signal processing techniques, lead to the compensation of some undesired effects on the sensors responses. Then, application in healthcare were discussed, where the POF sensors have been widely applied on wearable robot’s instrumentation and as wearable sensors for human movement assessment. In addition, the POF sensor technology has also enabled the development of many applications on other healthcare devices (such as SWs and systems for FES-assisted gait) and even in plantar pressure monitoring systems. As a last set of applications, the physiological parameters monitoring using POFs was discussed and its many applications were depicted. Thus, from simple light coupling systems to photonics textiles and integrated solutions, POF sensor technologies show many advantages and possibilities in healthcare. Compared with silica optical fibers, POFs have shown higher sensitivity and dynamic range due to their material features, where a higher strain limit result in a sensor with higher dynamic range than the ones of silica fibers and the lower Young’s modulus in conjunction with higher thermo-optic and thermal expansion coefficients resulted in sensors with higher sensitivity for physical parameters, where issues related to the polymer viscoelastic response can be overcame with proper signal processing techniques or thermal treatments. Despite their higher optical attenuation, POF sensors are suitable for short distance applications (commonly occurred in healthcare devices) and novel perfluorinated POFs resulted in systems suitable for operating even in the 1550 nm wavelength region. Therefore, it is possible to foresee an even higher popularization of such technology in the healthcare field, where some commercial initiatives beginning to appear, and the research of novel POF-based devices continue to grow. The next challenges to be overcame in POF technology are the issues related to the further miniaturization of the interrogation unit, especially for FBG sensors, where portables interrogators with wireless communication can further improve the sensor compactness and allow them to be integrated in even smaller structures. The integration of the POF sensors in smartphones for a portable and readily available interrogator is also a challenge for future studies, where, although they were already employed in intensity variation-based sensors [186], the smartphone application as interrogation unit for FBGs is a challenge. It is possible to expect that, once these challenges are overcome, the next generation of healthcare devices and wearable technologies will feature fully integrated sensors for portable solutions, where even the interrogation unit is incorporated on the devices with wireless data communication and direct control of wearable robots using POF sensors as their feedback information, creating a system with fiber-on-the-loop control.
